# Painless Loss of Vision as The First Presentation of Undiagnosed Neurofibromatosis 1 in A Child

**Published:** 2015

**Authors:** Siba Prosad PAUL, Shameem AHMED

**Affiliations:** 1 Specialty Trainee Year 8 in Paediatrics, Yeovil District Hospital, Yeovil, BA21 4AT, UK; 2 Consultant Neurosurgen, Gauhati MedicalCollege, Guwahati, 781032, India

**Keywords:** Neurofibromatosis type 1, Visual acuity loss, Optic pathway glioma, Annual ophthalmological assessment, MRI

## Abstract

**Objective**

A 10 year old presented with painless loss of vision as the first manifestation of neurofibromatosis 1 (NF1). Clinical assessment detected diagnostic features of NF1 and Magnetic Resonance Imaging (MRI) scan confirmed presence of plexiform neurofibroma and bilateral optic pathway glioma (OPG). The child was managed with chemotherapy which helped in improvement of vision. Review of current literature recommends vision testing in diagnosed cases of NP1 till 7 years of age; this is aimed at detecting visual impairments resulting from a symptomatic OPG.

## Introduction

Neurofibromatosis type 1 (NF1) is an inherited autosomal-dominant condition and is known to affect every organ system. In NF1, mutation (in NF1 gene located in chromosome 17q11.2) causes loss of neurofibromin which increases the risk of developing benign and malignant tumours(1). We report a case of a young child who presented with painless loss of vision with NF1 and emphasize the role of regular ophthalmological assessment.

**Case Study**

A 10-year-old boy presented to emergency department (ED) in a specialist hospital in north-eastern India with progressive painless dimness of vision in both eyes. Parents reported that that over the preceding 2 weeks he was finding it difficult to read and had deterioration in handwriting. He was repeatedly falling over and found it difficult to use the left side of his body. On arrival to the ED, observations were normal and Glasgow Coma Scale score was 15/15. Laboratory investigations were normal and his blood glucose was 86 mg/dl. General examination revealed multiple café au lait spots, axillary freckling and a soft tissue swelling over right infratemporal region (which was a neurofibroma). Neurological examination revealed decreased power of 4/5 (Medical Research Council scale) in left side with exaggerated reflexes. Vision checked in ED revealed 6/20 on both eyes. The pubertal assessment showed Tanner stage 2 (testes volume 4 ml, few pigmented sparse pubic hairs) but parents were not aware when the pubertal changes actually started. A clinical diagnosis of NF1 was made and he was admitted for neurological observations which remained stable. Magnetic resonance imaging (MRI) scan of brain was organised to investigate a possible intracranial space-occupying lesion. The MRI scan revealed a large plexiform neurofibroma in the right infratemporal fossa, bilateral optic pathway gliomas (OPG), parasellar lesion originating from hypothalamic region and hamartomas (unidentified bright objects) in pineal region. Brain biopsy was refused by parents after detailed explanation. The non-availability of biopsy results rendered the diagnosis of brain lesions likely to be OPG but not definitive ones and was managed in light of the clinical context of NF1. He did not have any siblings and his parents refused physical examination for themselves. Ophthalmology opinion was sought and the boy was transferred to the neurosurgeons. He underwent chemotherapy but parents did not agree for any surgical intervention. During the last follow-up his vision and left sided weakness had improved but has not completely normalized. He remains under care of the paediatric neurologists and ophthalmologists.

**Fig 1:**
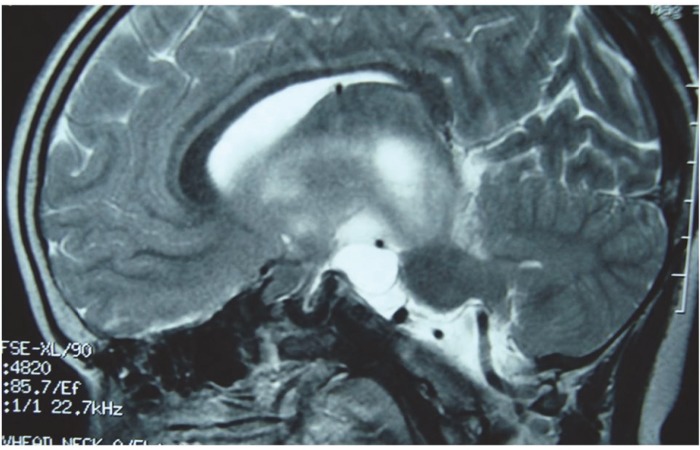
Sagittal section of MRI brain scan shows large mass in optic chiasmatic region suggestive of optic pathway glioma with hamartomas

**Fig 2:**
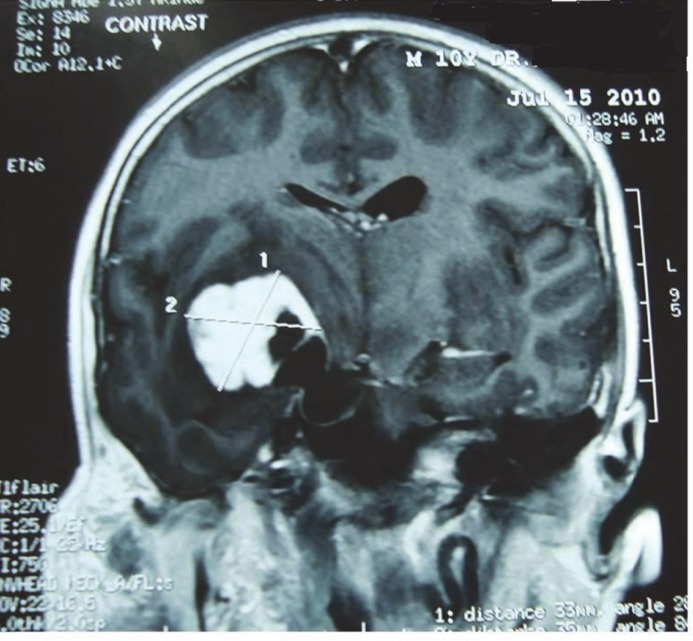
Coronal MRI scans of brain showing right parasellar mass

## Discussion

This case highlights the importance of considering NF1 in a child presenting with painless loss of vision. In developing countries especially in rural or semiurban settings, health infrastructure is aimed more at treatment of pathologies. In the absence of screening and monitoring for conditions such as NF1, different associations with NF1 will often present to the ED in specialist centers like the child described. Cases where delayed diagnosis of NF1 is made, it is important to elicit signs of precocious puberty (predominantly central in origin) and can be associated with OPG (2). OPGs occur in about 15% of children with NF1 and only 5% become symptomatic(1,3). A large number of OPGs remain asymptomatic for a long time before the manifestations caused by the tumours become evident: visual acuity loss, abnormal colour vision, visual field loss, squint, pupillary abnormalities, pale optic disc, proptosis and hypothalamic dysfunction(3). Young children under 7 years are at highest risk of developing OPGs(1) and are the most difficult age group to get an early diagnosis as they rarely complain of early visual impairment. The diagnosis of visual loss therefore remains difficult and is not often made until it becomes advanced with bilateral visual loss(3). Complete loss of vision due to OPG in children has been reported in the literature(4). The big question that the clinician faces is whether it is possible to predict the vision loss when presented with OPG. A study of 26 children with OPG(5) which included 47 study eyes (normal vision= 31, abnormal vision= 16); decreased macular ganglion cell layer-inner plexiform layer thickness was found to discriminate between children with and without vision loss from their OPG. Age-appropriate annual ophthalmological assessments in NF1 are recommended till 7 years of age in the UK(6). The US committee on genetics recommends intensive visual screening at 2, 4, 6, 9, 12, 15, 18, 24, 36, and 48 months of age(7). Visual-evoked potential testing has been found to detect OPGs at an earlier stage but is not available for routine clinical use currently(7). In developing countries, specialist pediatric ophthalmology services are available only in specialist centers and it is important to explain to parents the importance of regular visual monitoring in children with NF1. It is important to enquire about the child’s vision during assessment of children with NF1. Age-appropriate vision testing is recommended in all diagnosed cases of NF1 till the child reaches 7 years of age.
